# New Hampshire Journal of Medicine

**Published:** 1854-01

**Authors:** 


*New Hampshire Journal of Medicine.*—This contemporary has changed hands, a change made necessary, by the appointment of Dr. Edward H. Parker, its former editor, to a Professorship in a New York school. Dr. Geo. H. Hubbard now assumes the charge. We trust that he may find his labors both pleasant and profitable. S. B. H.

